# Transcriptome Analysis of the *Portunus trituberculatus*: De Novo Assembly, Growth-Related Gene Identification and Marker Discovery

**DOI:** 10.1371/journal.pone.0094055

**Published:** 2014-04-10

**Authors:** Jianjian Lv, Ping Liu, Baoquan Gao, Yu Wang, Zheng Wang, Ping Chen, Jian Li

**Affiliations:** 1 Key Laboratory for Sustainable Utilization of Marine Fisheries Resources, Ministry of Agriculture, Yellow Sea Fisheries Research Institute, Chinese Academy of Fishery Sciences, Qingdao, China; 2 College of Fisheries and Life Science, Shanghai Ocean University, Shanghai, China; State Key Laboratory of Pathogen and Biosecurity, Beijing Institute of Microbiology and Epidemiology, China

## Abstract

**Background:**

The swimming crab, *Portunus trituberculatus*, is an important farmed species in China, has been attracting extensive studies, which require more and more genome background knowledge. To date, the sequencing of its whole genome is unavailable and transcriptomic information is also scarce for this species. In the present study, we performed de novo transcriptome sequencing to produce a comprehensive transcript dataset for major tissues of *Portunus trituberculatus* by the Illumina paired-end sequencing technology.

**Results:**

Total RNA was isolated from eyestalk, gill, heart, hepatopancreas and muscle. Equal quantities of RNA from each tissue were pooled to construct a cDNA library. Using the Illumina paired-end sequencing technology, we generated a total of 120,137 transcripts with an average length of 1037 bp. Further assembly analysis showed that all contigs contributed to 87,100 unigenes, of these, 16,029 unigenes (18.40% of the total) can be matched in the GenBank non-redundant database. Potential genes and their functions were predicted by GO, KEGG pathway mapping and COG analysis. Based on our sequence analysis and published literature, many putative genes with fundamental roles in growth and muscle development, including actin, myosin, tropomyosin, troponin and other potentially important candidate genes were identified for the first time in this specie. Furthermore, 22,673 SSRs and 66,191 high-confidence SNPs were identified in this EST dataset.

**Conclusion:**

The transcriptome provides an invaluable new data for a functional genomics resource and future biological research in *Portunus trituberculatus*. The data will also instruct future functional studies to manipulate or select for genes influencing growth that should find practical applications in aquaculture breeding programs. The molecular markers identified in this study will provide a material basis for future genetic linkage and quantitative trait loci analyses, and will be essential for accelerating aquaculture breeding programs with this species.

## Introduction

*Portunus trituberculatus* (Crustacea: Decapoda: Brachyura), commonly known as the swimming crab, is widely distributed in the coastal waters of Korea, Japan, China, and southeast Asia [1]. This species inhabits estuaries and coastal waters, which belong to typical euryhaline crab species. In China, it is a major edible crab species and one of the most important fishery resources [2] and the production has now reached 92,907 tons2011 [3]. At present, Commercial crab farming largely depended on wild seed stock and the commercial characteristics (growth rate, flesh quality and disease resistance) of the cultured stocks have also declined after many years of culturing [4], and wild populations of *Portunus trituberculatus* have dramatically declined for the last decades due to over-exploitation and the deterioration of environmental conditions in China [5]. To improve the germplasm of swimming crab, a selective breeding for fast growing *Portunus trituberculatus* has been carried out since 2004 at the Yellow Sea Fisheries Research Institute, Chinese Academy of Fishery Sciences (Qingdao, China). The selected fast-growing population of the crab had been examined and approved by China National Aquaculture Variety Approval Committee as a new variety for aquaculture and named “HuangXuan No. 1” (Authorization number: GS-01-002-2012) in 2012, of which growth rate increased by 20.12% compared to wild seed stock.

However, the molecular mechanisms involved in growth are poorly understood. Due to a lack of genetic and genomic information, growth-determining genes have not been identified in the crab, even genes related to growth have rarely been reported. Therefore, more genome-wide or transcriptome-wide datasets should be generated as a basis for functional genomics approaches aimed at improving the aquaculture performance of this species. Despite the aquacultural and biological importance of *Portunus trituberculatus*, previous studies have mostly focused on the isolation of microsatellites [6]–[8], the investigation of genetic diversity [9], Sanger-based sequencing of expressed sequence tags (ESTs) [10], [11], the characterization of single functional genes [12]–[14] and sequencing of the mitochondrial genome of *Portunus trituberculatus* [15]–[17].

When no genome sequence is available, transcriptome sequencing is an effective way to obtain large numbers of molecular makers and identify transcripts involved in specific biological processes [18]. Massively parallel sequencing of RNA (RNA-Seq) has offered the opportunity to characterize the transcriptome with unprecedented sensitivity and depth. It has already revolutionized the way we study the transcriptome. The latest paired-end sequencing of RNA-Seq techniques have further improved the efficiency of DNA sequencing and expanded short read lengths, permitting a deeper understanding of the transcriptome [19]. Because it is not restricted by the unavailability of a genome reference sequence, this approach has been applied in decoding the genomes of several non-model organisms, providing valuable information in the understanding of gene function, cell responses and evolution [20]–[22]. Significant progress has aslo been made in understanding the transcript of various marine crustacea by RNA-seq over the last two years, such as *Litopenaeus vannamei*, *Fenneropenaeus chinensis*, *Eriocheir sinensis* and *Macrobrachium nipponense* [23]–[27], which is essential to better understand a species' biology and to devise strategies to improve productivity in culture. However, such investigations in *Portunus trituberculatus* have not been reported.

In this study, we present the first *Portunus trituberculatus* transcriptome using massively parallel mRNA sequencing. We perform Illumina sequencing of the eyestalk, gill, heart, hepatopancreas, and muscle tissues to characterize the *Portunus trituberculatus* transcriptome. The transcriptome provides an invaluable new data for a functional genomics resource and future biological research in *Portunus trituberculatus*. According to our sequence analysis, many genes involved in growth were identified. In addition, a variety of markers potentially useful for genomic population studies including simple sequence repeats (SSRs) located within coding regions and single nucleotide polymorphisms (SNPs) detected amongst deep coverage sequence regions reads are also reported.

## Materials and Methods

### Ethics Statement

The crabs used in the present study were marine-cultured animals, and all of the experiments were conducted according to the regulations of the local and central government.

### Sample Preparation

The swimming crabs, *Portunus trituberculatus* at 100 days age (20.62∼64.19 g in body weight), were obtained from a local farm in Qingdao, China. All the samples were acclimated in the laboratory (33 ppt, 18°C) for one week before the experiment treatment. The crabs (nine males and nine females) were all anaesthetized on ice and dissected to collect samples, including the eyestalk, gill, heart, hepatopancreas, and muscle. All of the samples were immediately in RNAlater (Ambion) at 4 °C overnight and then at −20 °C until RNA extraction within 2 weeks.

### RNA Isolation, cDNA Library Construction and Illumina Deep Sequencing

Total RNA was isolated from each sample by trizol (Invitrogen, CA,USA). RNA degradation and contamination was monitored on 1% agarose gels. RNA purity was checked using the NanoPhotometer spectrophotometer (IMPLEN, CA, USA). RNA concentration was measured using Qubit RNA Assay Kit in Qubit 2.0 Flurometer (Life Technologies, CA, USA). RNA integrity was assessed using the RNA Nano 6000 Assay Kit of the Bioanalyzer 2100 system (Agilent Technologies, CA, USA). A total amount of 5 ug RNA per sample was used as input material for the RNA sample preparations and all samples had RIN values above 8. Then, all samples were pooled in equal amounts to generate one mixed sample. The pooling samples were then used to prepare one separate Illumina sequencing libraries.

cDNA libraries were generated using Illumina TruSeq RNA Sample Preparation Kit (Illumia, San Diego, USA) following manufacturer's recommendations. Briefly, mRNA was purified from total RNA using poly-T oligo-attached magnetic beads. Fragmentation was carried out using divalent cations under elevated temperature in Illumina proprietary fragmentation buffer. First strand cDNA was synthesized using random oligonucleotides and SuperScript II. Second strand cDNA synthesis was subsequently performed using DNA Polymerase I and RNase H. Remaining overhangs were converted into blunt ends via exonuclease/polymerase activities and enzymes were removed. After adenylation of 3′ ends of DNA fragments, Illumina PE adapter oligonucleotides were ligated to prepare for hybridization. In order to select cDNA fragments of preferentially 200 bp in length, the library fragments were purified with AMPure XP system (Beckman Coulter, Beverly, USA). DNA fragments with ligated adaptor molecules on both ends were selectively enriched using Illumina PCR Primer Cocktail in a 10 cycle PCR reaction. Products were purified (AMPure XP system) and quantified using the Agilent high sensitivity DNA assay on the Agilent Bioanalyzer 2100 system. In the final step, the library preparations were sequenced on an Illumina Hiseq 2000 platform and 100 bp paired-end reads were generated.

### Availability of supporting data

The data sets of Illumina sequencing are being submitted to the NCBI Short Read Archive (SRA) database.

### Bioinformatic Analysis

#### Quality control

Raw data (raw reads) of fastq format were firstly processed through our self-written perl scripts. In this step, clean data(clean reads) were obtained by removing reads containing adapter, reads containing ploy-N and low quality reads from raw data. At the same time, Q20, Q30, GC-content and sequence duplication level of the clean data were calculated. All the downstream analyses were based on clean data with high quality.

#### Transcriptome assembly

Reads were assembled using Trinity [28], followed by TIGR Gene Indices clustering tools (TGICL) [29], with default parameters. The longest assembled sequences were referred to as contigs. The reads were then mapped back to contigs with paired-end reads to detect contigs from the same transcript and the distances between these contigs. Finally, sequences were obtained that lacked Ns and could not be extended on either end [30]. Such sequences were defined as unigenes.

#### Transcriptome annotation

The unigenes were predicted to mapping to protein-coding sequences by GetORF of EMBOSS [31]. The predicted protein-coding sequences were compared with the NCBI non-redundant (Nr) protein database and UniProtKB database using BLASTx with E values less than 1.0×10^−5^ (E values less than 1.0×10^−5^ were considered as significant) [32], [33]. Based on Nr annotation, we used BLAST2GO program (http://www.BLAST2go.org/) to get GO annotation of unigenes [34]. GO functional classification for all unigenes was performed using WEGO software (http://wego.genomics.org.cn/cgi-bin/wego/index.pl) [35]. KEGG metabolic pathway annotation and COG classification of unigenes were determined by BLASTx searching against KEGG database and COG database, respectively [36], [37].

#### Makers detection

SSR of the transcriptome were identified using MISA (http://pgrc.ipk-gatersleben.de/misa/misa.html), and primer for each SSR was designed using Primer 3 (http://primer3.sourceforge.net/releases.php). SNP were detected according to align clean reads to the reference transcriptome using SOAP2, then duplicated reads and multi-mapped reads were filtered from the alignment results in order to eliminate the PCR interference and ambiguous mapping. SOAPsnp was used to call SNP based on the sorted alignment results. SNPs qualified for the following standards were selected as the final SNP sets: quality score is not lower than 20, and distance between two SNPs are greater than 5.

### Real-time PCR Assays

14 annotated unigenes that may relate to growth were selected to be analyzed using real-time PCR, and their specific primers were listed in **Table S1**. The crab were siblings generated from a single pair of broodstock. The large and small sizes of crab were selected at 100 days age, respectively, from the >90 and <10 percentile regions of the growth distribution curve. Eyestalks were collected from nine healthy crabs of small size group (SG, 20.6±5.4 g in average body weight) and large size group (LG, 64.2±6.1 g in average body weight), respectively. All the samples were acclimated in the laboratory (33 ppt, 18°C) for one week before the experiment treatment. Total RNA was isolated according to the manufacturer's instructions of TRIZOL LS reagent (Invitrogen, Carlsbad, CA, USA). Then, RNA samples of three individuals were pooled within each group in equal amounts to generate three mixed sample, respectively. (three biological replicates of each group). RpL8 gene was selected as an internal control for qPCR analysis and the primers reference Xu's literature [38]. First strand cDNA was synthesized from 1 mg of RNA using M-MuLV reverse transcriptase (Qiagen). The qPCR reaction mixture (20 uL) consisted of 26 Power SYBR Green PCR Master mix, 0.9 M each of the forward and reverse primers, and 1 mL of template cDNA. PCR amplification was performed under the following conditions: 50°C for 2 min and 95°C for 30 s, followed by 40 cycles of 95°C for 15 s and 62°C for 1 min, and a final extension at 72°C for 5 min.

### SSR validation and Polymorphism evaluation

Genomic DNA of crabs was extracted from muscle tissue using genomic DNA extraction kit (BioTeke, Beijing, China) following the protocols. Electrophoresis through a 1.5% agarose gel was used to check DNA integrity. The SSR markers were initially tested for amplification using a pool DNA sample of 10 crabs. PCR amplifications were carried out using Master-cycler gradient thermal cycler (Eppendorf) in a final volume of 10 ul. Each reaction tube contains 1.0 μl of 10×PCR buffer, 0.8 μl of dNTP (2.5 mM), 0.4 μl of each primer (10 umol), and 0.5 μl of genomic DNA (20 ng/ul), 0.05 μl of rTaq DNA polymerase (5 U/ul,Takara), 6.85 uL of ddH_2_O. The PCR reaction program was: DNA denaturation at 94°C for 5 min; 35 cycles of 94°C for 30 s, 50–60°C for 30 s, 72°C for 30 s.; and 72°C for 7 min as a final extension. The primers that were not successful for amplification or produced multiple bands were reanalyzed using the touchdown PCR method with 1°C increments. The optimized SSR primers were used to amplify DNA from 30 wild individuals of *P. trituberculatus* collected from Jiaozhou, Shandong province, China for polymorphism evaluation. Amplification products were resolved via 8% denaturing polyacrylamide gel, and visualized by silver-staining. A 10-bp DNA ladder (Invitrogen Inc.) was used as a reference marker for allele size determination.

The number of alleles (*Na*), polymorphism information content (PIC), expected and observed heterozygosities (*He* and *Ho*, respectively) were calculated with the software CERVUS 3.0 [39].

### SNP validation

To validate the putative SNPs identified in transcripts, the same cDNA samples as for the transcriptome profiling (pool of eighteen wild *Portunus trituberculatus*) were used. Twenty transcripts containing 56 potential SNPs and sufficient flanking regions were randomly selected for primer design. PCR products were sequenced directly in both directions with forward and reverse primers using Sanger technology on the ABI3730 platform (Applied Biosystems). Sequencing chromatograms were visually analyzed with Chromas2.32 (Technelysium Pty. Ltd.), and SNPs were identified as overlapping nucleotide peaks.

## Results and Discussion

### Illumina Draft Reads and Sequence Assembly

**Figure 1:**
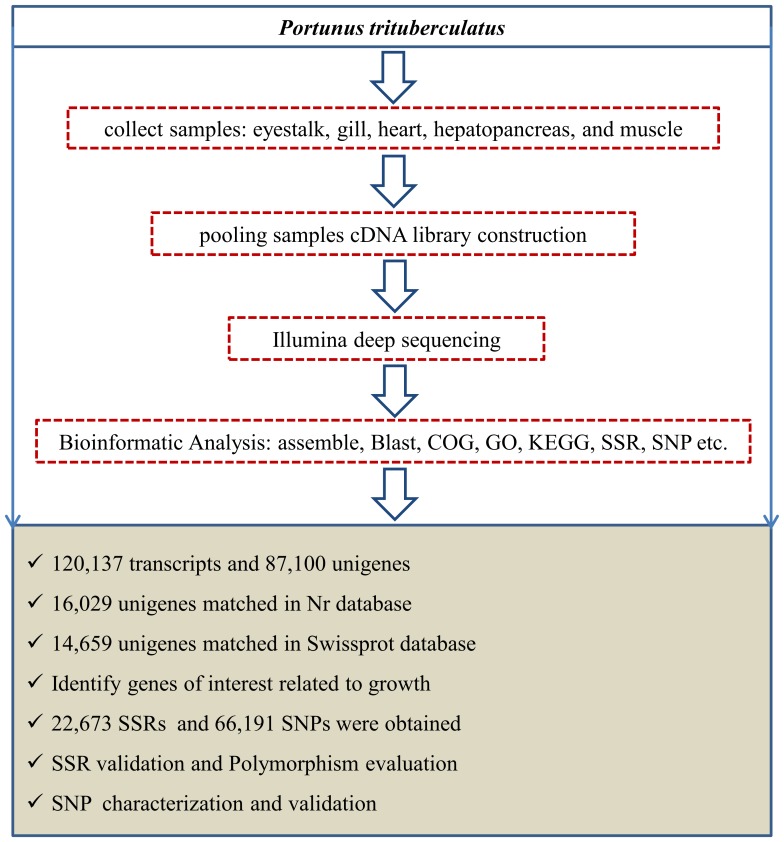
Schematic of Illumina deep sequencing and analysis. It includes sample preparation, cDNA library construction and Illumina sequencing, data analysis including assemble, blast, GO annotation, SSR and SNP analysis, etc.

In order to achieve a comprehensive *Portunus trituberculatus* transcriptome, total RNA was extracted from a variety of tissues, including the eyestalk, gill, heart, hepatopancreas, and muscle. Equal quantities of RNA were mixed together to construct a cDNA library and perform Illumina sequencing. This pooling strategy was widely used in some similar studies [23], [24], [40], [41]. The schematic of Illumina deep sequencing and analysis are shown in Figure 1. The overall Illumina read pairs and clean bases for all samples are 65,846,872 and 12.86G, respectively (**Table 1**). Files containing these data were deposited in the Short Read Archive of the National Center for Biotechnology Information (NCBI) with accession numbers of SRR1168416 and SRR1168417.

**Table 1:** Summary of Illumina transcriptome sequencing, assembly and annotation for *Portunus trituberculatus*.

Raw results (after trimming)		Assembly results		Annotation results	
Clean bases (G)	12.86	Transcripts(bp)	120,137	Nr annotations	16,029
Read pairs	65,846,872	Average length of transcripts (bp)	1,037	UniProtKB annotations	14,659
Read length (bp)	100	Smallest transcripts (bp)	201	COG hits	14,263
		Largest transcripts (bp)	33,865	GO mapped	26,732
				KEGG hits	7,588

**Figure 2:**
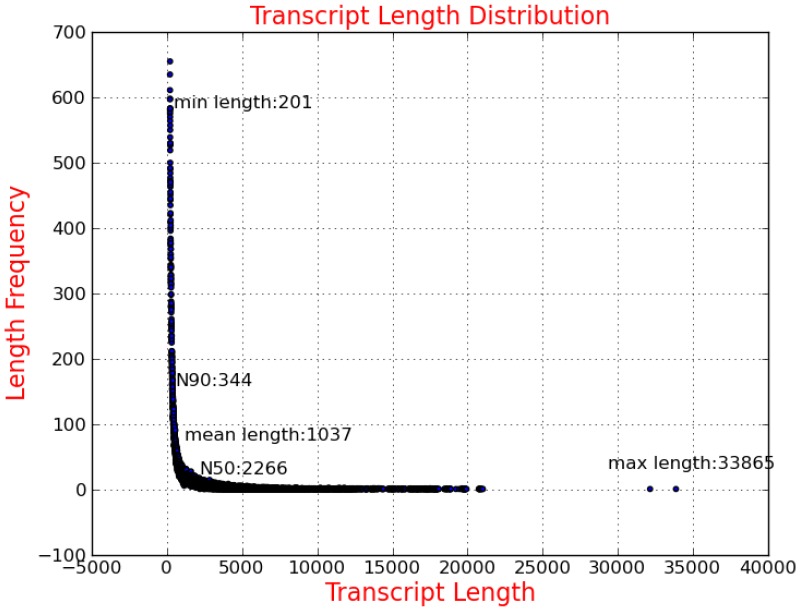
Sequence length distribution of transcripts assembled from Illumina reads.

After assembly analysis based on all Illumina reads, we identified 120,137 transcripts. The average length of all transcripts was 1,037 bp, with the smallest sequence of 201 bp and the largest one of 33,865 bp. The sequence length distribution of transcripts is indicated in **Figure 2** and **Table 1**. The average length of our assembled contigs was longer than that previously reported for *Litopenaeus vannamei* (average of 396 bp), *Eriocheir sinensis* (average of 385 bp) and *Fenneropenaeus chinensis* (average of 676 bp) [24], [25], [42]. Long sequences of good quality could enable us to obtain more information about genes. Therefore, this transcriptome dataset provides a useful resource for future analyses of genes related to economic traits. To the best of our knowledge, this is the first comprehensive study of the transcriptome in *Portunus trituberculatus*.

To assess the abundance and coverage of the transcriptome data, we matched the assembled unigenes against the known EST library on Genbank. The 13,985 ESTs downloaded from NCBI were clustered and assembled, and 2,612 assembled EST-unigenes with mean length of 783 bp were generated. Comparisons between transcriptome unigenes and EST-unigenes were performed using BLASTn algorithm. All of the EST-unigenes can be matched in the transcriptome unigenes library, whereas only 3.0% of the transcriptome unigene sequences can be found in the ESTs library. It suggests the transcriptome data provide abundant information besides the now available ESTs sequences, and will vastly expand the number of genes identified in this species.

### Annotation of Unigenes

After ruling out short-length and low-quality sequences, 87,100 unigenes were selected and subjected to annotation analysis by matching sequences against Nr and UniProtKB databases using BLASTx searching with an E value 1.0×10^−5^. 16,029 unigenes (18.40% of the total) can be matched in Nr database, and 14,659 (16.83% of the total) matched in UniProtKB (**Table 1** **and Table S2**). A significant number of *Portunus trituberculatus* unigenes did not matching any sequences in the GenBank nr database which is not surprising for crustacean transcriptome studies [26], [43]–[46]. Whilst most of these likely represent transcripts spanning only untranslated mRNA regions, chimeric transcript sequences derived from assembly errors or transcripts containing non-conserved protein regions, as reported in other transcriptome analyses [47]–[49], it is also possible that some may constitute novel genes unique to this species.

**Figure 3:**
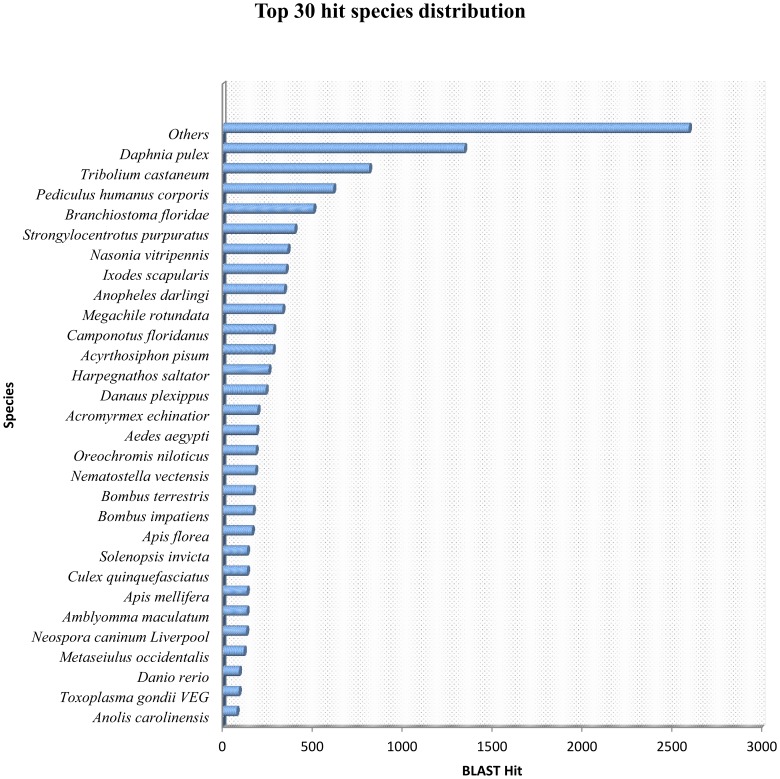
Top 30 hit species distribution based on BLASTx.

For main species distribution matched against Nr database, 9.58% of the matched unigenes showed similarities with *Daphnia pulex*, a microcrustacean, whose draft genome was recently published, followed by *Tribolium castaneum* (5.81%), *Pediculus humanu scorporis* (4.39%), *Branchiostoma floridae* (3.60%), *Strongylocentrotus purpuratus* (2.85%), *Nasonia vitripennis* (2.58%), *Ixodes scapularis* (2.50%), *Anopheles darlingi* (2.43%), *Megachile rotundata* (2.37%), *Camponotus floridanus* (2.00%), *Acyrthosiphon pisum* (2.00%)), and others (18.5%). As might be expected, unigenes of transcriptome matched well to crustacean and other arthropod proteins (**Figure 3**) which are in agreement with previous crustacean studies [26], [43]–[46].

### COG, GO and KEGG Classification

The assembled unigene sequences were subjected to BLAST searching against GO, COG and KEGG databases, and the summary statistics of BLAST assignment was shown in**Table 1**.

**COG** is a database where orthologous gene products were classified. Every protein in COG is assumed to be evolved from an ancestor protein, and the whole database is built on coding proteins with complete genome as well as system evolutionrelationships of bacteria, algae and eukaryotes [50]. Phylogenetic classifications of the predicted CDSs of unigenes were analyzed by searching against COG database to predict and classify possible functions of the unigenes (**Figure S1**). Possible functions of 14,263 unigenes were classified and subdivided into 26 COG categories, among which the cluster for‘General function prediction only’ represents the largest group (2652, 16.38% of the matched unigenes), followed by ‘Signal Transduction’ (2,279, 14.08%) and ‘Posttranslational modification, protein turnover, chaperones’ (1,483, 9.16%).

**The Gene Ontology (GO)** project provides structured, controlled vocabularies and classifications that cover several domains of molecular and cellular biology and are freely available for community use in the annotation of genes, gene products and sequences. Many model organism databases and genome annotation groups use the GO and contribute their annotation sets to the GO resource [51]. Among 87,100 assembled unigenes, 26,732 were successfully annotated by GO assignments, belonging to one or more of the three categories: biological process, cellular component, and molecular function. Among the annotated unigenes, 18,517 are involved in various biological process categories, cellular process (13,977 unigenes; 16.05%), metabolic process (12,657; 14.53%), biological regulation (4,824; 5.54%) and regulation of biological process(4,612; 5.30%) comprised the largest proportion. Further, 15,405 unigenes are involved in cellular component categories, among which, cell part (13,174; 15.12%), cell (13,174; 15.12%), organelle (5,712; 6.56%) and macromolecular complex (3,748; 4.30%) comprised the largest proportion. In addition, 21,988 unigenes are involved in molecular function categories, the top four categories were involved in binding (14,155; 16.25%), catalytic activity (10,779; 12.38%), transporter activity (2,467; 2.83%) and structural molecule activity (1,776; 2.04%) (**Figure S2**). In summary, these terms account for a large fraction of the overall assignments in *Portunus trituberculatus* transcriptomic dataset. Understandably, genes encoding these functions may be more conserved across different species and are thus easier to annotate in the database.

**The KEGG pathway** database records networks of molecular interactions in the cells and variants of them specific to particular organisms. Pathway-based analysis helps us to further learn biological functions of genes [36], [52]. To systematically analyze their inner cell metabolic pathways and complicated biological behaviors, we classified the unigenes into biological pathways by mapping the annotated CDS sequences to the reference canonical pathways in the KEGG database (**Figure S3**). 7,588 unigenes were consequently assigned to 31 KEGG pathways, among which 858 members assigned to ‘Translation’, followed by ‘Signal Transduction’ (818 members), ‘Folding’ (750 members), ‘Carbohydrate Metabolism’ (699 members), ‘Nervous System’ (560 members), ‘Transport and Catabolism’ (542 members), Cell Growth and Death’ (510 members), ‘Replication and Repair’ (493 members), Acid Metabolism’ (475 members), ‘Immune System’ (458 members), ‘Nucleotide Metabolism’ (431 members) and others.

GO, KEGG pathway and COG analysis are helpful for predicting potential genes and their functions at a whole-transcriptome level. The predicted GO categories, metabolic pathways, together with the COG analysis, are useful for further investigations of gene function in future studies.

### Genes of interest related to growth

The transcriptome of *Portunus trituberculatus* was primarily examined to identify a wide range of candidate genes that might be functionally associated with growth. Traditionally, such gene discovery in non-model organisms has required degenerate PCR, which is labor-intensive and prone to failure [53]. The annotated transcriptome reported here allows researchers to identify genes of interest more easily than that of using degenerate PCR, especially in crustaceans whose genome information is relatively poor. According to our sequence analysis, many genes involved in growth were identified (**Table 2**) via three principal search strategies [54]: 1) associations between genes and growth reported in crustaceans, 2) growth-related genes involved with moulting, 3) muscle development and degradation genes involved in moulting.

**Table 2:** Genes of interest for growth and muscle development in *Portunus trituberculatus*.

Candidate genes	Transcript IDs Contig IDs
**5-Hydroxytryptamine receptor**	comp2095_c0;comp2005_c0;comp580344_c0;
**Alpha-amylase**	comp57976_c0;comp45223_c0;
**Cathepsin L**	comp459050_c0;comp14425_c0;comp31871_c0;comp31366_c1;comp11733_c0;comp14990_c0;comp38911_c0;comp722731_c0;comp27256_c0;
**Cyclophilin**	comp437375_c0;comp423352_c0;comp499501_c0;comp289233_c0;comp9973_c0;comp530052_c0;comp59112_c0;comp682946_c0;comp50416_c0;
**Fatty acid-binding protein**	comp31398_c0;
**Fibrillarin**	comp41046_c0;comp48390_c0;
**Profilin**	comp41014_c0;comp39876_c0;comp33022_c0;comp51876_c0;
**Growth hormone and insulin-like growth factor**	comp56179_c4;comp22818_c0;comp388372_c0;comp430291_c0;comp14260_c0;comp38363_c1;comp49319_c0;comp38363_c0;comp380665_c0;comp552_c0;comp46854_c0;comp514057_c0;comp55694_c0;comp58751_c0;
**Myostatin and growth differentiation factor 8/11**	comp623701_c0;comp317115_c0;
**SPARC**	comp45527_c0;comp49996_c0;
**Ecdysteroid**	comp31811_c0;comp30741_c0;comp56215_c0;
**CHH**	comp15032_c0;comp196993_c0;
**Gonad/vitellogenesis-inhibiting hormone (G/VIH)**	comp342264_c0;comp51889_c0;comp57850_c2;comp508364_c0;
**Methyl farnesoate and farneosoic acid O-methyltransferase**	comp17060_c0;comp55531_c1;comp48156_c0;
**MIH**	comp198837_c0;
**Actin**	comp31467_c0;comp58899_c0;comp48501_c1;comp54937_c0; comp57254_c0; comp87442_c0; comp378707_c0
**Myosin**	comp427727_c0;comp381601_c0;comp45465_c1;comp60783_c0;comp45934_c0;comp30670_c0;comp39835_c0;comp51426_c0;comp46623_c0;comp23652_c0;comp129272_c0;comp31352_c0;comp31357_c1;comp33150_c0;comp40995_c0;
**Alpha skeletal muscle**	comp50088_c0;comp324557_c0;comp45287_c0;
**Calponin/calponin transgelin**	comp41232_c0;comp52466_c0;comp57804_c0;
**Tropomyosin**	comp48547_c0;comp59654_c0;
**Muscle lim protein**	comp59750_c0;comp60108_c0;comp59060_c0;comp48583_c0;comp31515_c0;

In this study, a total of 21 categories of growth-related genes were found, amongst these, ten were found in our transcriptome data which have been identified previously to have roles in growth in crustaceans. These genes include 1. 5-Hydroxytryptamine receptor, Alpha-amylase, Cathepsin L, Cyclophilin, Fatty acid-binding protein, Fibrillarin, Glyceradehyde-3-phosphate dehydrogenase, Growth hormone and insulin-like growth factor, Myostatin and growth differentiation factor 8/11, Signal transducer and activator of transcription, Secreted protein acidic and rich in cysteine(SPARC), and Translin-associated factor X (TRAX). Previous studies have shown that SNP in 5-Hydroxytryptamine receptor [55], Cathepsin L [56], Myostatin and growth differentiation factor 8/11 genes [57] show significant associations with growth traits in crustaceans. Besides, Cyclophilin, Fibrillarin and Secreted protein acidic and rich in cysteine(SPARC) gene expression showed a negative correlation with body weight in shrimp by Pearson's correlation analysis [58].

In crustaceans, periodic shedding of the exoskeleton is one of the most important physiological processes essential for crustacean growth and postembryonic development including moulting and regeneration [59]. Although the functions of many of the hormones and genes involved in this process are still not well defined, a number of studies have indicated that moulting and reproduction in crustaceans is regulated by the eyestalk derived CHH gene family which is one of the major groups of peptide hormones produced in the XO-SG [60], [61]. In addition, MIH is responsible for maintaining animals in the intermoult stage which is an important regulator of steroidogenesis in the YO [62]. In this research, a few genes which belong to crustacean hyperglycemic hormone neuropeptides (CHH) family were identified including MIH, CHH and ecdysteroids. Correlations between SNPs in the CHH and MIH gene with individual growth performance show that the CHH and MIH gene has high potential to impact body weight variation in crustaceans [63] and should, therefore, be considered as a primary gene of interest in growth studies.

Crustacean muscle growth is not continuous and is strongly influenced by the moulting cycle [59]. During the moult, muscles regenerate, and energy reserves including glycogen and lipids are accumulated in the hemolymph and the midgut for the next moult [64], [65]. Overall muscle protein synthesis is very important for growth, reproduction and other metabolic activities in crustaceans. Recent studies of invertebrates have highlighted the importance of muscle specific genes and proteins in crustaceans [43]. In this research, six genes related to muscle build-up or degradation during the moulting event were identified including Actin, Myosin, Alpha skeletal muscle, Calponin/calponin transgelin, Tropomyosin and Muscle lim protein. Among which, both actin and myosin proteins showed a high number of transcripts. It has been reported that actins are expressed in abundance as they are critical to formation of muscle filaments [66]. Different actin isoforms have been identified in various crustaceans [67], and are likely to be involved in playing important roles in cytoskeletal structure, cell division and mobility, and muscle contraction [68], [69]. As evidence for a role for actin in muscle build-up during the moult cycle in crustaceans, Cesar and Yang (2007) reported that muscle structural α-actin and cytoskeletal β-actin increased during the intermoult and premoult stages, a phase where high muscle growth occurred in the abdominal muscle of L. vannamei [70]. However, in a recent SNP association analysis study, four synonymous polymorphisms were identified in an actin fragment but SNP allele distributions were not related significantly to individual growth performance in the two studied groups of giant freshwater prawn *M.rosenbergii* [63], and further studies will be required to investigate. Myosins are a major component of the contractile apparatus and consist of two heavy (MHC) and four associated light chains (MLC) [71]. Previous study showed, myosin gene expression levels could provide a good molecular marker of individual growth potential in the Atlanticpink shrimp *Farfantepenaeus paulensis* that identified MHC as a possible growth candidate gene [72]. A high number of actin and myosin protein transcripts observed here may regulate muscle development and function in *Portunus trituberculatus*, and similar results have been found in *Macrobrachium rosenbergii* [43], however, further studies are needed to confirm these observations.

The current study identified a number of putative genes that are potentially involved with growth in *Portunus trituberculatus*. However, further studies are needed to understand the molecular functions of these putative genes with growth performance.

### Real-time RT-PCR confirmation of growth-related genes

To futher confirm the growth-related genes obtained from the transcriptome data, 14 candidate genes were selected to be analyzed using real-time PCR. Since the XO-SG complex in the crab eyestalk produces a variety of neuropeptides/neurohormones [60], [61], it was selected, in this study, as the target tissue for differential gene expression analysis between large and small crab.

**Figure 4:**
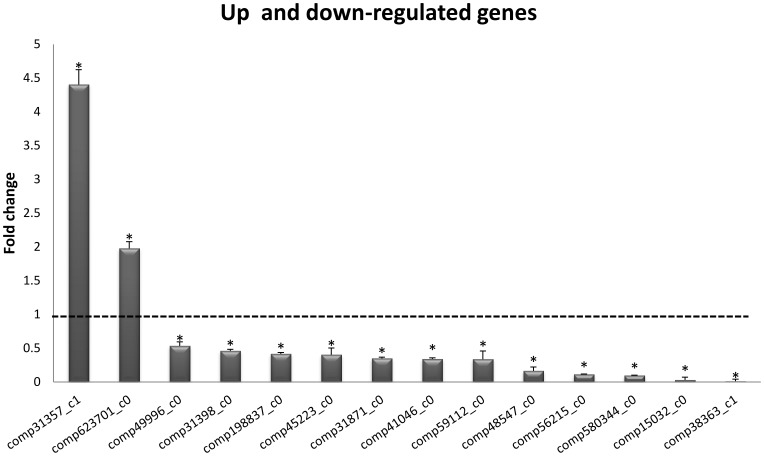
Relative expression of 14 growth-related genes (LG Vs SG). Real-time PCR was performed on template cDNA from LG and SG (three biological replicates of each group). The dotted line indicates expression of the SG group, columns indicate expression of the LG group, normalized to reference gene RpL8. Significant up- or down-regulation in LG indicated with an asterisk (P<0.05).

All of the 14 selected genes revealed significant differences in gene expression between small size group (SG) and large size group (LG) (**Figure 4**), which consistent with the fact that body weight is a complex trait regulated by the coordinate action of several genes. Most of gene (12) were significantly down-regulated in LG including cyclophilin A (comp59112_c0), fibrillarin (comp41046_c0) and SPARC (comp49996_c0). In *P. monodon*, Tangprasittipap et al. (2010) reported the index of relative cyclophilin, SPARC and fibrillarin-like expression was negatively correlated with body weight (p<0.05) [58], which were similar to our results, suggests that these genes may have some effect on individual growth performance, and warrants further study in crustacean species.

Myostatin (comp623701_c0) and Myosin heavy chain (comp31357_c1) were up-regulated in LG. In vertebrates, Myostatin (MSTN), principally controls growth of muscle cells as a negative regulator of muscle development [73], however, MSTN show positive regulation of growth in the crab. Similar result was found in *P. monodon*, reduced levels of MSTN transcripts resulted in a dramatic slowing of growth rate compared with control groups [74], which suggests the genes may regulate growth positively in crustaceans. Myosins are a major component of the contractile apparatus and consist of two heavy and four associated light chains [71]. In south-western Atlantic pink shrimp *Farfantepenaeus paulensis*, higher MHC expression was observed in a high weight shrimp group [72], a result was similar to our research, which suggested MHC as a possible growth candidate gene in crustaceans.

The result here show that seeking the growth-related genes via high-throughput transcriptome sequencing and bioinformatics analysis is an effective way. Further studies should be carried out to elucidate the function of these genes in growth.

### Putative molecular markers

#### SSR characterization and Polymorphism evaluation

SSRs, or microsatellites, are polymorphic loci present in genomic DNA. They consist of repeated core sequences of 2∼6 base pairs in length. Among the various molecular markers, SSRs have been proven to be an efficient tool for constructing genetic linkage, performing QTL analysis and evaluating the level of genetic variation in a species because of the high variability, abundance, neutrality and co-dominance of microsatellite DNA [75].

**Figure 5:**
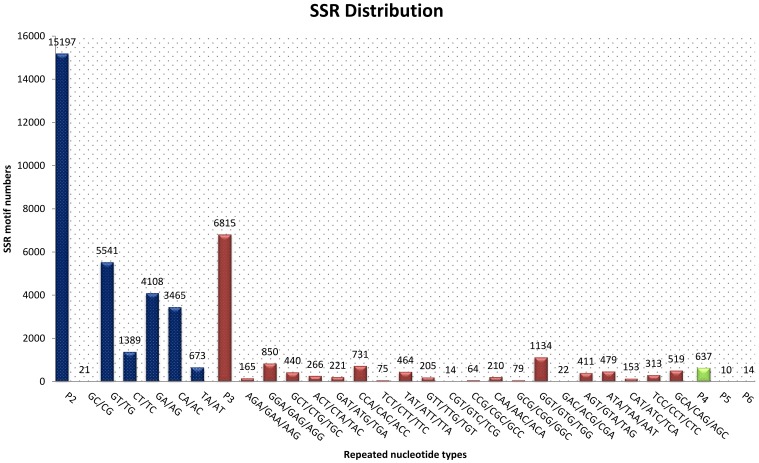
Distribution of simple sequence repeat (SSR) nucleotide classes among different nucleotide types found in *Portunus trituberculatus*.

We obtained a total 22,673 SSRs in the transcriptomic dataset. Of these, 67.03% were di-nucleotide repeats, followed by 30.06% tri-nucleotide repeats and 2.92% tetra/penta/hexa-nucleotiderepeats (**Figure 5**). Generally believed that SSR of animals are mainly di-nucleotide repeats [76], [77], and our findings support this conclusion. Among the di-nucleotide repeats motifs, (GT/TG)n, (GA/AG)n and (CA/AC)n were the three predominant types with frequencies of 36.46%, 27.03% and 22.80%, respectively. In the 20 types of tri-nucleotide repeats, (GGT/GTG/TGG)n, (GGA/GAG/AGG)n, and (CCA/CAC/ACC)n were the most common types with a combined frequency of 39.84%. The results of this study is differ from other studies which indicate that microsatellite repeats types have species-specific in crustacea [26], [43]. Primer for each SSR was designed using Primer 3 (http://primer3.sourceforge.net/releases.php), and 14.5% (3,858) SSRs can be designed priemers successfully (the data do not show). To date, only a few microsatellites have been available for *Portunus trituberculatus* from NCBI. Thus, the development of SSRs for this species is highly desirable.

**Figure 6:**
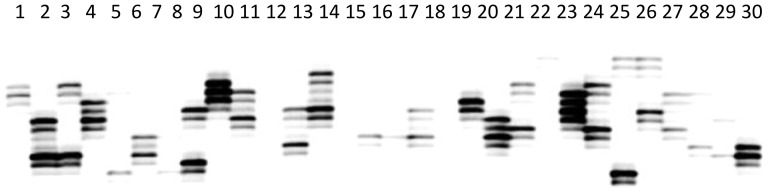
Polyacrylamide gel electrophoresis for one SSR markers (comp17478_c0) in the 30 individuals.

Fifteen SSRs were randomly selected for primer synthesis and validation, among which, 9 were successful in PCR amplification using genomic DNA from *Portunus trituberculatus*. The remaining 6-pair primers failed to generate PCR products, even when the annealing temperature was reduced by 8°C. Of the 9 primers, 1 primers were monomorphic, the other 8 primers were polymorphic (**Figure 6**), the proportion of polymorphic primers was 53.3%. From the 8 polymorphic loci, the number of alleles per locus ranged from 2 to 8 alleles. A total of 34 alleles were identified, with an average of 4.25 alleles per locus. Across 8 loci, the polymorphic information content (PIC) ranged from 0.15 to 0.78 (**Table 3**), with an average of 0.58, suggesting that the developed EST-SSRs were highly polymorphic. The results obtained in this study indicated that these SSRs developed from EST in the swimming crab will be a useful tool for the genetic research such as population variation, parentage analysis, stock enhancement evaluation, and the establishment of effective conservation strategy of *Portunus trituberculatus*.

**Table 3:** Characterization of 15 polymorphic microsatellite loci in *Portunus trituberculatus*.

Transcript IDs	(Type) No.	Primer sequence (5′-3′)	Length	*Ta*	*Na*	*Ho*	*He*	PIC
**comp57884_c0**	(TG)9	F: TTGGAAGTTTTGTTGGCGGC	199	59	3	0.59	0.64	0.56
		R: ACGTTGTGGTCACTTGTTGC						
**comp32503_c0**	(TG)9	F: GCGGCGTGGTAATCTCTATGT	199	60	4	1	0.74	0.70
		R: CTAACAAGACATCCACGCACG						
**comp333871_c0**	(TG)8	F: GCAGGGGCTTCTTCACCTTA	235	60	5	0.96	0.79	0.74
		R: CCCCTCCTTCCAATTTGTACCT						
**comp31578_c0**	(CA)8	F: GACCAACAGGCGACCGAG	134	57	2	0.2	0.18	0.16
		R: AGCCGCTTCCCGAGATTC						
**comp49316_c0**	(TC)9	F: TGCGTTGCGTAACTGAGTCT	269	59	4	1	0.63	0.56
		R: CTTGTCAGTGCTTCCTTGCC						
**comp55771_c0**	(TG)10	F: AAGACCCCGAGGAAGAGC	199	55	5	1	0.75	0.69
		R: GCAAGGCATATCCAACAT						
**comp54398_c4**	(TC)9	F: CTTTGTGTGTTGGGTTGGGC	142	58	3	0.72	0.55	0.45
		R: AACAATGCCCACAACTCAGG						
**comp17478_c0**	(GT)10	F: GCATAGAACGAGTGATACTAGATGC	235	59	8	0.92	0.79	0.78
		R: CACACACACACACACACACA						
**comp53876_c0**	(GT)10	F: ACAGCGTCAGGAAGCAATCA	209	60	1	0	0	0
		R: ATTGCCTCCTTCCCAATGCA						
**comp49119_c1**	(CA)9	F: TGGTTTTGCCACTCCACACT	182	59	n/a			
		R: TGTCAGCCACGACACTACTT						
**comp44386_c0**	(CA)7	F: TTGCGTGTGGAGGAAGTGTT	277	60	n/a			
		R: GAGAGGTGGATGGGGAGACT						
**comp16699_c0**	(AG)7	F: TTCTCATTCTTGGCTCGCGT	205	60	n/a			
		R: TCACATGCTCCGAGGATTGG						
**comp24638_c0**	(AG)8	F: CGTGTGTGCGTGTTTGTCTT	279	58	n/a			
		R: AGTTTTCCTCCTTTACGCATCA						
**comp58479_c1**	(TG)6	F: AGGACCATAACAAGGCCACG	176	60	n/a			
		R: CTGCAACACAGCACTGACAG						
**comp1580_c0**	(TG)7	F:CACCCAAGCTCTCTTCCTGG	228	59	n/a			
		R: ACCAACAGACAGGGAGAGGA						

*Ho* observed heterozygosity, *He* expected heterozygosity, *Na* observed number of alleles, *Ta* annealing temperature, PIC polymorphic information content, n/a indicates that no PCR amplification.

#### SNP characterization and validation

**Figure 7:**
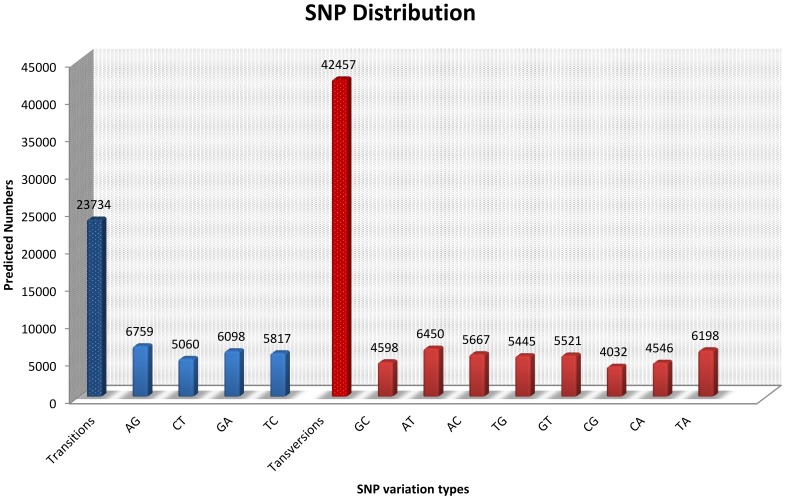
Distribution of putative single nucleotide polymorphisms (SNP) in *Portunus trituberculatus* sequences.

SNPs were identified from alignments of multiple sequences used for contig assembly. By excluding those that had mutation frequency of bases lower than 1%, we obtained a total of 66,191 SNPs, of which 23,734 were putative transitions (Ts) and 42,457 were putative transversions (Tv), giving a mean Ts: Tv ratio of 1∶1.79 across the transcriptome of *Portunus trituberculatus* (**Figure 7**) which can help identify genes affected by selection [78]. Further analysis found the Ts: Tv ratio were species-specific in crustacean species, in Oriental River Prawn (*Macrobrachium nipponense*) and Giant Freshwater Prawn (*Macrobrachium rosenbergii*), the Ts: Tv ratios were 1.99∶1.00 and 1.32∶1.00, respectively. The AT/TA, AG/GA and CT/TC SNP types were the most common. In contrast, GC/CG types were the smallest SNP types because of the differences in the base structure and the number of hydrogen bonds between different bases [26].

To verify the potential SNPs, a subset of 20 transcripts containing 56 SNPs were selected randomly. A pooled cDNA sample of eighteen wild *Portunus trituberculatus* was amplified by PCR. Subsequently, PCR products were sequenced bidirectionally with forward or reverse primers (**Table S3**), among which, sequencing of four transcripts failed. Of the 43 SNPs predicted to reside in the amplified sequences, 21 (48.8%) showed polymorphisms in the sample and were validated (**Table S3**). The rate of polymorphic SNPs was probably an underestimate because only eighteen individuals were used. In addition, the way of SNP validation in this study has some limitations, SNPs with smaller variation frequency were difficult to identify via overlapping nucleotide peaks. Consequently, using more *Portunus trituberculatus* samples, the polymorphic rate of potential EST-SNPs should be higher than that found in our validation.

SSRs and SNPs detected in this study (**Table S4 and Table S5**) are likely to be highly transferable to other closely related species, as has been the case in other crustacean species [79], [80]. These potential markers identified within the ESTs will be valuable for studying the evolution and molecular ecology, genome mapping, and QTL analysis of *Portunus trituberculatus*.

## Conclusion

Here we report the first comprehensive transcript dataset of the *Portunus trituberculatus*, a non-model species for which little molecular knowledge currently exists. The 120,137 transcripts identified and assembled will enable gene discovery in *Portunus trituberculatus*, and with the significant number of putative growth-related genes identified should facilitate genomics approaches to improving the growth performance of domesticated stocks used for aquaculture. In addition, the large number of SNPs and SSRs detected provide targets for identifying polymorphisms across *Portunus trituberculatus* populations useful for parentage assignment and for managing in breeding in cultured populations.

## Supporting Information

Figure S1 **COG classification of the unigenes.**
(PNG)

Figure S2 **GO classification of all unigenes.**
(PNG)

Figure S3 **KEGG classification of the unigenes.**
(PNG)

Table S1 **The primers of Real-time PCR of growth-related genes.**
(DOCX)

Table S2 **Summary of annotation results for transcripts of** ***Portunus trituberculatus*** **.**
(XLS)

Table S3 **Primers used and verified SNPs in the transcripts of** ***Portunus trituberculatus*** **.**
(DOCX)

Table S4 **Summary of putative SNPs from** ***Portunus trituberculatus*** **.**
(XLSX)

Table S5 **Summary of putative SSRs from** ***Portunus trituberculatus*** **.**
(XLSX)
