# Supplementary figures and images for: Transcriptome Analysis of the Portunus trituberculatus: De Novo Assembly, Growth-Related Gene Identification and Marker Discovery
Source: PLoS One. 2014 Apr 10;9(4):e94055. doi: 10.1371/journal.pone.0094055 (PMC3983128; doi:10.1371/journal.pone.0094055)

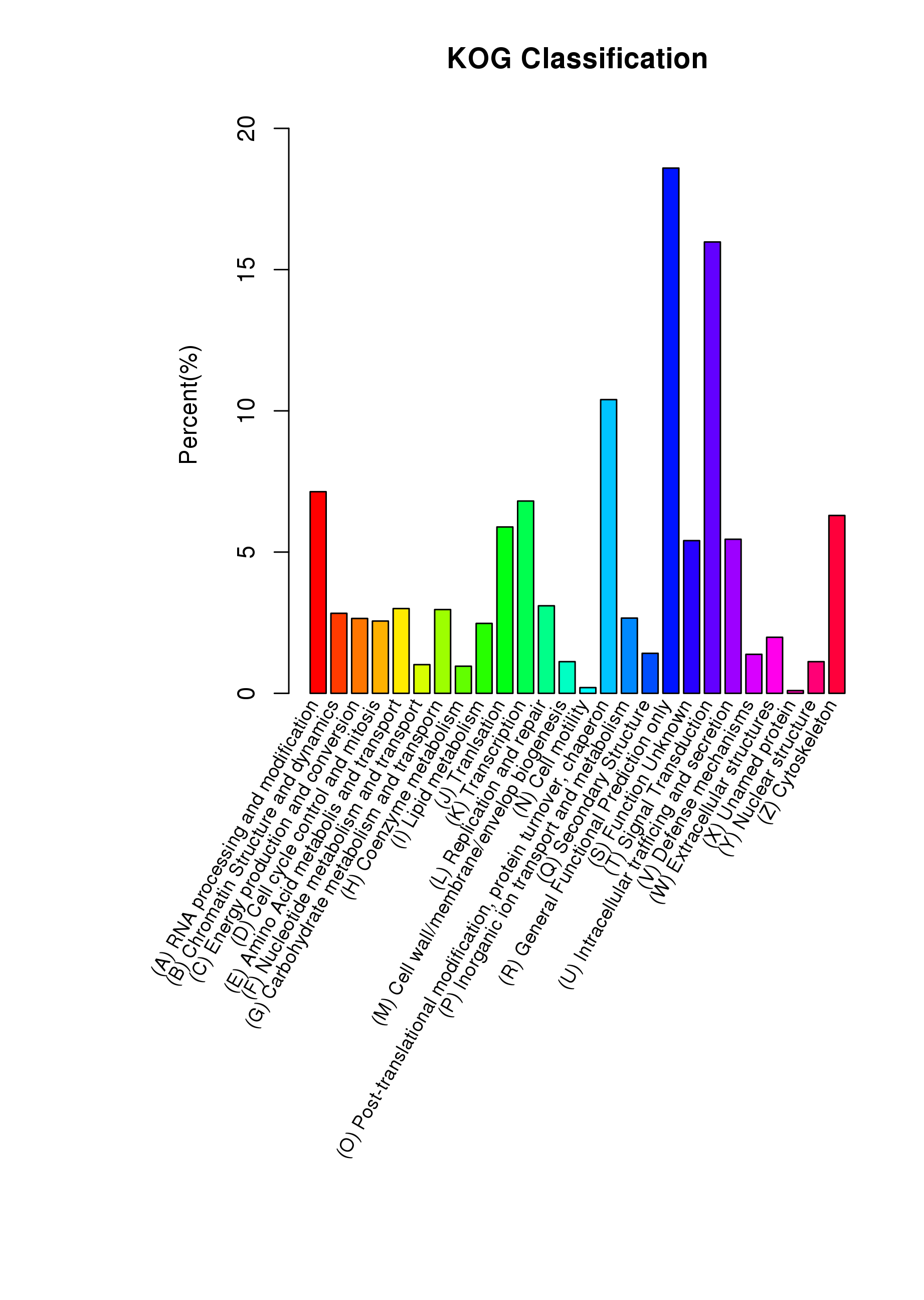

Supplement: Figure S1 — COG classification of the unigenes. (PNG) [file pone.0094055.s001.png]

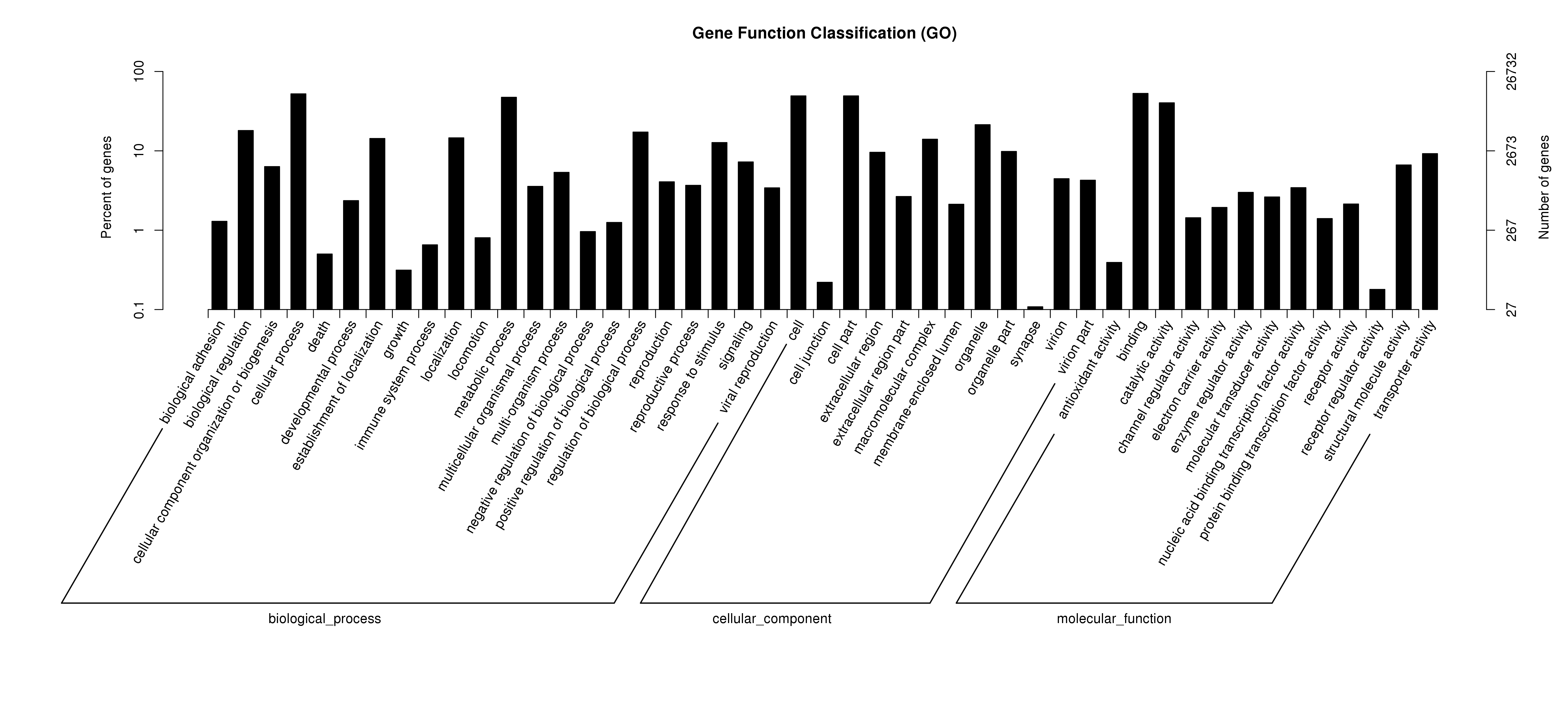

Supplement: Figure S2 — GO classification of all unigenes. (PNG) [file pone.0094055.s002.png]

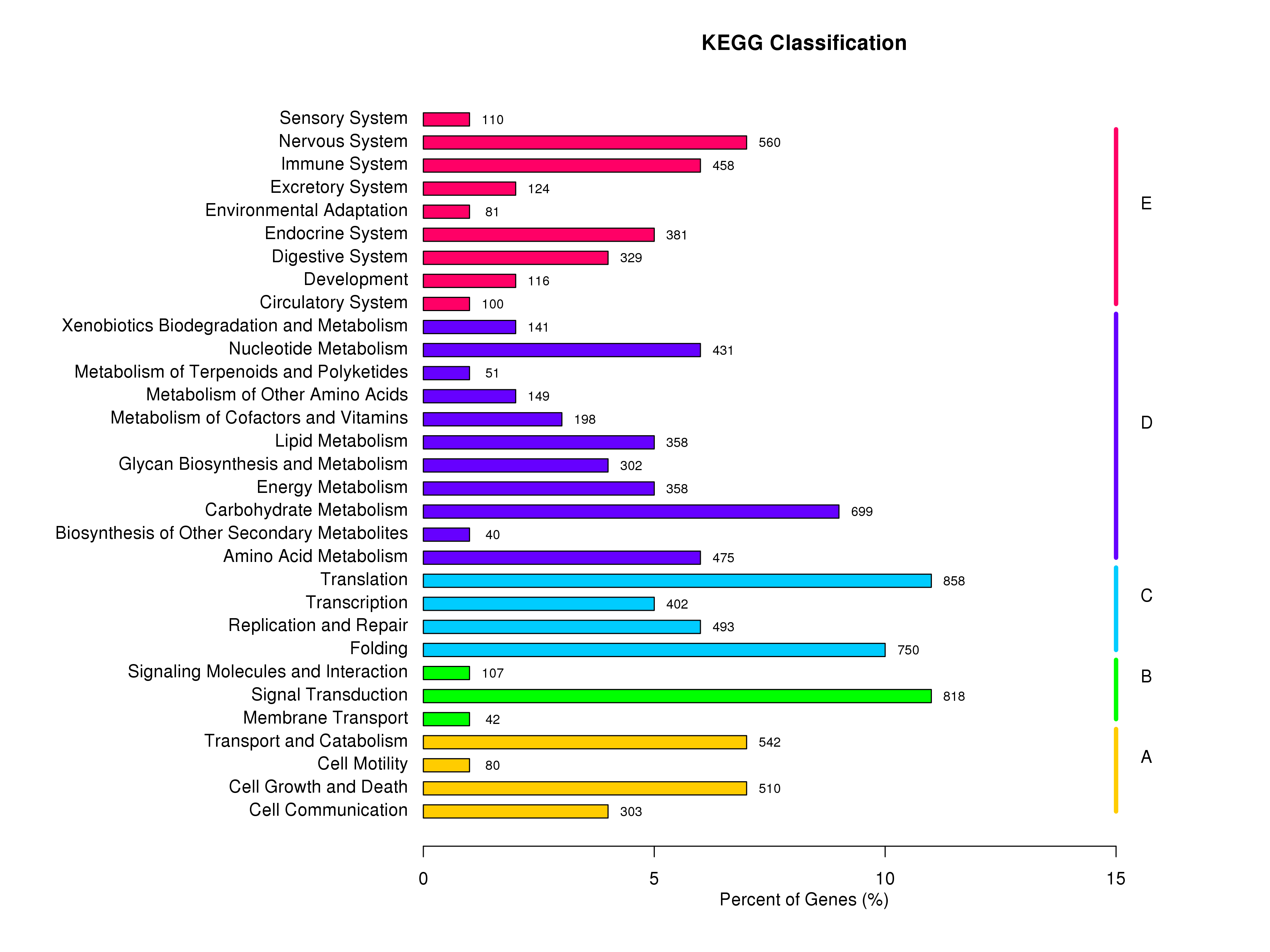

Supplement: Figure S3 — KEGG classification of the unigenes. (PNG) [file pone.0094055.s003.png]
