# Supplementary material for: Transcriptome Analysis of the Portunus trituberculatus: De Novo Assembly, Growth-Related Gene Identification and Marker Discovery
Source: PLoS One. 2014 Apr 10;9(4):e94055. doi: 10.1371/journal.pone.0094055 (PMC3983128; doi:10.1371/journal.pone.0094055)
Supplement: Table S1 — The primers of Real-time PCR of growth-related genes. (DOCX) [file pone.0094055.s004.docx]

**Table S1: The primers of Real-time PCR of growth-related genes**

| Transcript IDs | Gene name | Primer sequence (5'-3'） |
| --- | --- | --- |
| comp15032_c0 | CHH | F:GTCTGCACGATCTGGTCCTCAAGGTTCT |
|  |  | R:GGTTATTGCCTACTTCGCCAATGTCGGT |
| comp198837_c0 | MIH | F:GTTCGCCTCCACCACTACCACCATCA |
|  |  | R:GGAATACCAACAGCAGCCAGCCACTC |
| comp31357_c1 | myosin heavy chain | F:TGCCCTAATTGCTATCAGTTGGAGATGC |
|  |  | R:GGACAGCACTACGCCCGTCATGTTAG |
| comp31398_c0 | fatty acid-binding protein | F:AGTGTGGCTCCTGTTGGTCCTTCTCT |
|  |  | R:GGTGGAACAGTCAATCAGGTTCTGCTCT |
| comp31871_c0 | cathepsin L | F:CGGTACTGAGCGGCTGGTCTGTGTT |
|  |  | R:ACTTTCGGAGTTGGCGTCCCATGTTTG |
| comp38363_c1 | insulin-like receptor | F:TGATGATTGAGCCTCACCGTCACGAG |
|  |  | R:CACCATAGACACTTTCTCCCACCACCAT |
| comp41046_c0 | fibrillarin | F:GCCGCCGCATGATGACAACTACAAC |
|  |  | R:CAACGGTGTTCGCAAATCCATCCATTCC |
| comp45223_c0 | alpha amylase | F:CCCGTGCTGTAAACTTCTCACCGTCTG |
|  |  | R:CCTGGTCCATCTCATCGGCAATGTTCTT |
| comp48547_c0 | Tropomyosin | F:ACCAGACTTACTCCTCACGAATGCCTTC |
|  |  | R:ACCAACGACGACCACGCCATCA |
| comp49996_c0 | SPARC | F:AGCGAAGATTGGAGAGTTGGTGACCGA |
|  |  | R:CAGAGTGTGATGGTGTGGGCGTGAC |
| comp56215_c0 | Ecdysone receptor | F:GAGAGGCTTGTTGTCGGCAGAGATGTC |
|  |  | R:CGAGTCGTCAAGGCGTGCAGTTGT |
| comp580344_c0 | 5-hydroxytryptamine receptor 1 | F:GGACACTCGCCGTTGCAGAAGTTAGC |
|  |  | R:TGCCGCTACCACCTTACCGTCAACT |
| comp59112_c0 | cyclophilin A | F:TTCAGCGAGTTGGAGCATGTC |
|  |  | R:AAGTTGTCCATCAGCAGCAGG |
| comp623701_c0 | Myostatin | F:GCAGTTGAAACAGTGGGTGAGG |
|  |  | R:CCAGTATCGGTGTGAGATTCAGC |
