# Supplementary material for: Transcriptome Analysis of the Portunus trituberculatus: De Novo Assembly, Growth-Related Gene Identification and Marker Discovery
Source: PLoS One. 2014 Apr 10;9(4):e94055. doi: 10.1371/journal.pone.0094055 (PMC3983128; doi:10.1371/journal.pone.0094055)
Supplement: Table S3 — Primers used and verified SNPs in the transcripts of Portunus trituberculatus . (DOCX) [file pone.0094055.s006.docx]

**Table S3. Primers used and verified SNPs in the transcripts of *Portunus trituberculatus.***

| Transcript IDs | Primer sequence (5'-3'） | Products length（bp） | No. of SNPs |
| --- | --- | --- | --- |
| comp27308_c1 | AATGGTATTGGCGTCGCTC | 672 | 1/3/0 |
|  | CATATTTGGAAACCGTGCTGG |  |  |
| comp31118_c0 | AATGATCAAACAAAGTTTTACAATACAC | 698 | 1/1/0 |
|  | ACCCTCCAAATATGGCCAAG |  |  |
| comp31535_c0 | ATATTAAGTTGCGTCCAGACAAG | 705 | 5/3/2 |
|  | CGAAATTACACTTAAAACTCCTG |  |  |
| comp32722_c0 | CTTGATGACATGGCAAGGATC | 815 | 0/1/0 |
|  | GGACAAGCTGGATGGCTAGTG |  |  |
| comp32727_c0 | AATGATCGCCAGTCCACTACAC | 947 | 0/1/0 |
|  | AAACTTGATGCACAGTCTTACAAGTG |  |  |
| comp53176_c0 | GAGGAGGAGGAAGAACACTAACAG | 622 | 1/5/0 |
|  | ATGAAGGTGGTGGCAATGG |  |  |
| comp54165_c0 | TCAATTATGCATGTGATGTCGTT | 875 | 3/5/0 |
|  | CTGGCAGGAGTAAGTCATCTGTACT |  |  |
| comp54166_c0 | GTGTTCCGCTGCTTCATCTG | 967 | n/a |
|  | CGCTGAGTTCCAGGAAGACTGAC |  |  |
| comp54621_c0 | CACAACAAGTTGAGTACATTGTCG | 908 | 1/1/0 |
|  | GGTGGATACGCATGTGACG |  |  |
| comp85434_c0 | GGTGGTACTCTTCCTCCTCG | 835 | n/a |
|  | ACATGACATGTACACGTCCATTAG |  |  |
| comp45967_c0 | TTTGCCTCTGCTCCTAAGTAAG | 888 | 6/3/3 |
|  | GTCTGTAGCAACATTCACAAGGT |  |  |
| comp59116_c0 | AAACTGGAAGTAGCAGACTGATCT | 916 | n/a |
|  | TGCATTGAGACGTATGGAGATAG |  |  |
| comp30889_c0 | AAACCTACTGGTGTTACATGCAC | 856 | n/a |
|  | GCCAAGTAATGAGATAAGTTGCC |  |  |
| comp49307_c0 | GGAGGACGTACAATAACTGCC | 803 | 2/3/0 |
|  | CAGGTTGAGGTCACAGAGCC |  |  |
| comp49564_c0 | TTCAATGTCCTCAGCCAAGTT | 806 | 1/1/0 |
|  | TGTCTAATACGCTTCCTCCTCC |  |  |
| comp31541_c0 | CTTGGTTCTCGGTGAGTCACC | 844 | 2/5/0 |
|  | GCCTTGCAGAAGAATCTTGAC |  |  |
| comp40789_c0 | AATAGCCATCTGAAATACTAGG | 923 | 0/1/0 |
|  | AAATTGACAGACAGTGTCCC |  |  |
| comp40896_c0 | CAACCTTAGCAAGTTTAATGCTGC | 911 | 2/4/0 |
|  | AGTCAGACTGGTCCATGACAACG |  |  |
| comp51894_c0 | CAATCATGAAGACTCTTGTACTCTGC | 789 | 1/2/0 |
|  | CAGTTCATACTGTATTGGCAGTAATC |  |  |
| comp57612_c2 | CACTGTTACTTCATTGCGGG | 721 | 1/1/0 |
|  | GAGGGAATATTGAAAGACTAGGG |  |  |

For each transcript, the following are indicated: the number of SNPs verified/the number of SNPs predicted in the fragment/the number of additional SNPs using Sanger technology; n/a indicates that sequencing was not possible because the sequencing signal was interrupted.
